# Clinical and Genetic Features of Biallelic Mutations in *SORD* in a Series of Chinese Patients With Charcot-Marie-Tooth and Distal Hereditary Motor Neuropathy

**DOI:** 10.3389/fneur.2021.733926

**Published:** 2021-11-08

**Authors:** Xiaoxuan Liu, Ji He, Mubalake Yilihamu, Xiaohui Duan, Dongsheng Fan

**Affiliations:** ^1^Department of Neurology, Peking University Third Hospital, Beijing, China; ^2^China-Japan Friendship Hospital, Beijing, China; ^3^Key Laboratory for Neuroscience, Ministry of Education/National Health Commission, Peking University, Beijing, China; ^4^Beijing Municipal Key Laboratory of Biomarker and Translational Research in Neurodegenerative Diseases, Beijing, China

**Keywords:** Charcot-Marie-Tooth disease, phenotype, genotype, genetics, distal hereditary motor neuropathy, *SORD*

## Abstract

Biallelic mutations in the *sorbitol dehydrogenase (SORD)* gene have recently been found to be one of the most frequent causes of autosomal recessive axonal Charcot-Marie-Tooth (CMT2) and distal hereditary motor neuropathy (dHMN). This study was performed to explore the frequency of *SORD* mutations and correlations of the phenotypic-genetic spectrum in a relatively large Chinese cohort. In this study, we screened a cohort of 485 unrelated Chinese patients with hereditary neuropathy by using Sanger sequencing, next generation sequencing, or whole exome sequencing after *PMP22* duplication was initially excluded. *SORD* mutation was identified in five out of 78 undiagnosed patients. Two individuals carried the previously reported homozygous c.757 delG (p.A253Qfs^*^27) variant, and three individuals carried the heterozygous c.757delG (p.A253Qfs^*^27) variant together with a second novel likely pathogenic variant, including c.731 C>T (p.P244L), c.776 C>T (p.A259V), or c.851T>C (p.L284P). The frequency of *SORD* variants was calculated to be 6.4% (5/78) in unclarified CMT2 and dHMN patients. All patients presented with distal weakness and atrophy in the lower limb, two of whom had minor clinical sensory abnormalities and small fiber neuropathy. Our study provides further information on the genotype and phenotype of patients with *SORD* mutations.

## Introduction

Charcot-Marie-Tooth disease (CMT) is a clinically and genetically heterogeneous group of inherited neuropathies that share common characteristics of distal muscle wasting, sensory loss, and decreased deep tendon reflexes (1). CMT can be classified as demyelinating (CMT1) and axonal (CMT2) according to motor nerve conduction velocity (MCV) in the upper limb nerve. CMT is also closely related to distal hereditary motor neuropathies (dHMNs), which manifest only as motor involvement with or without minor sensory involvement. Two disorders are sharing many causative genes (e.g., *HSPB1, HSPB27, BSCL*_2_*, DCTN1*) (2), and they represent a continuum from pure motor neuropathy to motor and sensory neuropathy (1). To date, >100 genes have been identified to be related to hereditary neuropathies. The detection rate for causal mutations among CMT2 and dHMN patients is between 20 and 30%, indicating that more than half of patients remain undiagnosed (3).

Since biallelic mutations in the *sorbitol dehydrogenase (SORD)* gene were *first* described in May 2020, 14 mutations have been identified to our knowledge (4–7). *SORD*-related neuropathy has been reported as one of the most frequent causes of autosomal recessive CMT2 and dHMN (4). The prevalent mutation is c.757delG (p.A253Qfs^*^27), and almost all previously reported mutations in patients with CMT and dHMN are related to this variant, either in homozygous or heterozygous states (4, 5, 7), except one Chinese patient with dHMN harboring the compound heterozygous c.404 A>G and c.9081 + G>C mutation (6). Most of the mutations in *SORD* are frameshift or splicing mutations, indicating a loss of function of sorbitol dehydrogenase, which is a key enzyme that converts sorbitol to fructose. The pathological mechanism by which *SORD* mutations cause motor-predominant peripheral neuropathy is not well understood.

In this study, we screened *SORD* mutations in a cohort of 78 Chinese patients with unclarified CMT2 and dHMN and 650 healthy controls. We aimed to assess the clinical and genetic features of *SORD* mutations in the Chinese population.

## Patients and Methods

### Patients

We enrolled 485 unrelated Chinese patients with hereditary neuropathy in Peking University Third Hospital and China-Japan Friendship Hospital between January 2007 and December 2020. The patients were classified based on their clinical phenotypes, mode of inheritance, and electrophysiological features. The family history, age of onset, clinical features, CMT neuropathy score (CMTNS), and electrophysiological features of the patients were also recorded in detail. All patients were evaluated by at least one skilled neurologist. The study was approved by the Ethics Committee of Peking University Third Hospital (IRB 00006761). Written informed consent was obtained from the patients or their parents for the publication of this report and any accompanying images.

### Mutation Analysis

**Figure 1:**
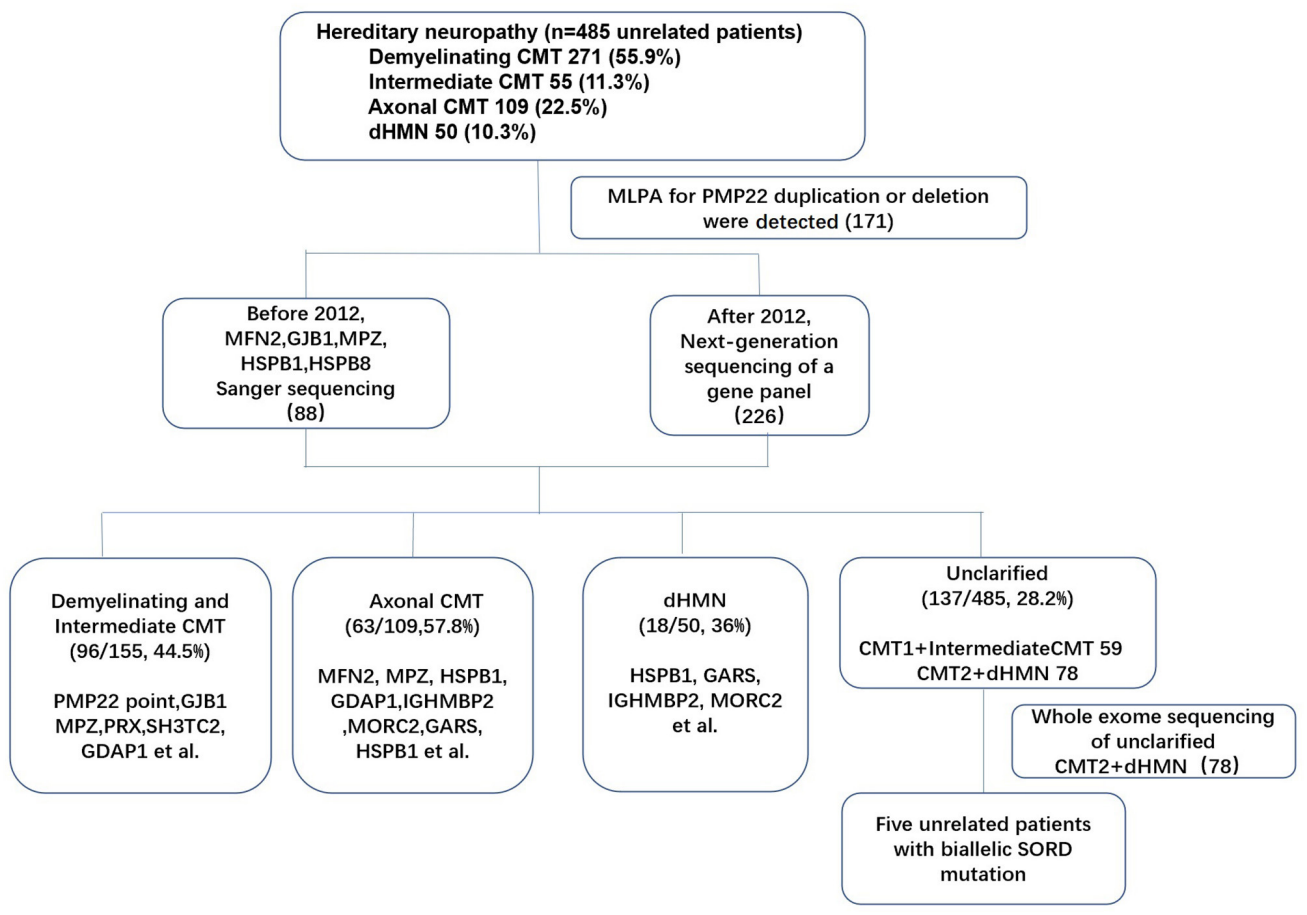
Genetic testing flow chart for patients with hereditary neuropathy in our study.

Genomic DNA was extracted from the peripheral blood of the subjects using a DNA isolation kit (Bioteke, AU1802). Concentrations were determined with a Qubit fluorometer (Invitrogen, Q33216) and a Qubit dsDNA HS assay kit (Invitrogen, Q32851). Agarose gel (1%) electrophoresis was performed for quality control. The multiplex ligation-dependent probe amplification (MLPA) technique was applied in all patients with demyelinating and intermediate CMT, and 171 index patients with *PMP22* duplications were initially excluded. After performing Sanger sequencing (before 2012) or next generation sequencing (NGS) of a gene panel (after 2012) in the remaining patients (314 index patients), 78 patients with CMT2 and dHMN remained undiagnosed. Then, whole-exome sequencing (WES) was performed for 78 index patients. Sample dilution, flow cell loadings, and sequencing were performed according to the Illumina specifications. DNA libraries were sequenced on the HiSeq X10 (Illumina, San Diego, CA) as paired-end 150-bp reads. (The flow chart of genetic testing is illustrated in Figure 1). All suspected variants were validated by Sanger sequencing of *SORD*. All nine *SORD* coding exons (NM_003104) were amplified by polymerase chain reaction. The amplicons were analyzed using an ABI 3730XL DNA analyzer (Applied Biosystems, Waltham, MA) in accordance with the manufacturer's protocol.

### Pathogenicity Prediction

The pathogenicity of *SORD* mutations was assessed using phenotypic characterization; screening in the database dbSNP identifiers (http://www.ncbi.nlm.nih.gov/projects/SNP), 1,000 genomes (http://1000genomes.org/), and GnomAD comparison with 650 Chinese healthy controls; and *in silico* pathogenicity prediction by SIFT (http://sift.jcvi.org/www/SIFT_enst_submit.html), PolyPhen, and Mutation Taster (http://www.mutationtaster.org/). Variant classification was based on American College of Medical Genetics (ACMG) standards (2015) (8), and pathogenic or likely pathogenic variants were identified accordingly.

## Results

### Clinical Features

Of the 485 unrelated patients with hereditary neuropathy in our cohort, 271 had received a clinical diagnosis of CMT1, 109 had CMT2, 50 had dHMNs (pure dHMN and motor-CMT2) and 55 had intermediate CMT families. *SORD* mutations on both alleles were detected in five of 78 patients with unclarified CMT2 (46) and dHMN (32) (Figure 1). Of the 78 patients, there was AD inheritance in 15, AR inheritance in 8, and sporadic diseases in 55. All of the five patients with biallelic *SORD* mutations were sporadic without family history. The frequency of *SORD* mutations was calculated to be 1% (5/485) in all hereditary neuropathy patients and 6.4 % (5/78) in patients with unclarified CMT2 and dHMN.

All patients developed their first symptom in childhood or adolescence (Table 1). The mean age of onset was 14 years, with a range from 6 to 17 years. The mean disease duration was 16 years, with a duration range from 7 to 30 years, and the progression rate was mild or moderate, as evidenced by a mean CMTNS of 12.2 (range of 9–15). Foot drop, walking difficulties, and pes cavus were present in all affected patients. They shared a common phenotype, with motor-predominant distal muscle weakness and atrophy of the lower limb and decreased or absent deep tendon reflex and foot deformities. Only two patients (patients 1 and 5) had mild weakness in the distal upper limb, with an MRC score of 5- in the intrinsic muscle (Table 1). Numbness and episodic pain of the distal limb and a positive pinprick test were observed in patients 1 and 5; thus, these two patients with minor sensory involvement were classified as having CMT2, and the other three cases were consistent with dHMN. All patients maintained the ability to walk without assistance throughout the entire follow-up period.

**Table 1:** Clinical and electrophysiological features of the patients in this study.

	**1.1406**	**2.1924**	**3.1414**	**4.0803**	**5.1008**
Mutation	c.757delG/c.757delG	c.757delG/c.757delG	c.757delG/c.776 C>T	757delG/c.731 C>T	757delG/c.851 T>C
Mode of inheritance	Sporadic	Sporadic	Sporadic	Sporadic	Sporadic
Onset age (year)	17	6	16	15	16
Sex	F	F	M	F	M
Disease duration (y)	15	30	12	7	16
Phenotype	dHMN	dHMN	dHMN	dHMN	dHMN
Muscle strength					
APB/FDI/ADM	5-/5-/5–	5/5/5	5/5/5	5/5/5	5-/5/5
Dorsiflexion/plantar flexion	1/2	2/3	2/3	3/3	3/3
Sensory test					
Pinprick test	↑ Up to knee	-	-	-	↑ Up to knee
Vibration	-	-	-	-	-
DTR (UL/LL)	++/+	++/-	+/-	-/-	+/-
CMTNS	15	9	14	11	11
Nerve conduction velocity					
Median nerve					
CMAP (mV)	11.5	6.1	11.2	6.4	3.1
MCV (m/s)	61.2	45.2	52.1	47.1	48.5
Peroneal nerve					
CMAP (mV)	0.2	0.8	1.2	Abs	3.4
MCV (m/s)	42.3	47.6	47.9	Abs	50.7
Sural nerve					
SNAP (μV)	17.8	10.3	8.6	7.5	15.6
SCV (m/s)	57.4	52.8	48.7	49.2	51.6

*Muscle strength: according to MRC score*.

*APB, abductor pollicis brevis; FDI, first dorsal interosseous; ADM, abductor digiti minimi*.

*Sensory test: ↑ increased sensation; - negative*.

*DTR: deep tendon reflex; tendon reflex statuses: +++ active; ++ normal; + reduced; - absent*.

*CMTNS, Charcot-Marie-Tooth neuropathy score*.

*CMAP, compound muscle action potential; MCV, motor conduction velocity; SNAP, sensory nerve action potential; SCV, sensory conduction velocity*.

*The abnormal value of the CMAP and MCV of the motor nerve is marked in red*.

**Figure 2:**
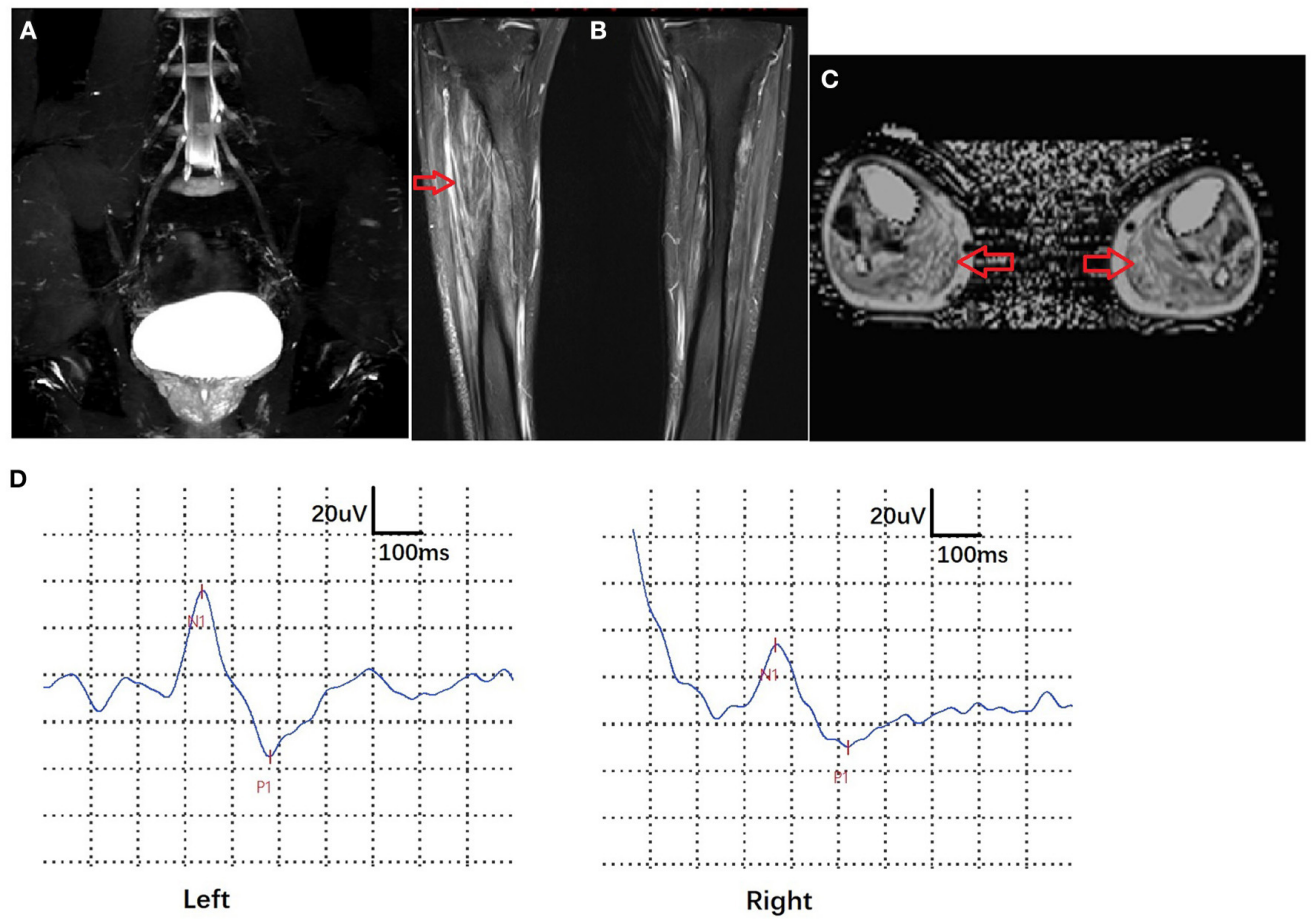
Imaging and electrophysiological features of patient 5. MRI of lumbar nerves (coronal 3D-STIR) showed normal morphology of lumbar nerve roots and lumbar plexuses. **(A)** MRI of calf skeletal muscles (T2WI FS cor, fat fraction) exhibited slight fatty infiltration and edema in the bilateral anterior tibial muscle and extensor hallucis longus and distinct fatty infiltration in the gastrocnemius muscle (arrows) **(B,C)**. Contact heat evoked potential (CHEP) showed a prolonged latency (366 vs. 337 ms) and decreased amplitude (43.5 vs. 70.8 μV) on the right side of the leg vs. the left **(D)**.

Electrophysiological studies revealed pure motor axonal neuropathy in all individuals with biallelic mutations in *SORD*. The decrease in compound muscle action potential (CMAP) tended to be more severe in the lower compared with the upper limbs. CMAP of the peroneal nerve was absent in one of the five patients. The mean CMAP was 1.9 mV (range, 0–5.3 mV) for the peroneal nerve and 7.7 mV (range, 3.1–11.5 mV) for the median nerve. Motor nerve velocity was within the normal range in both the upper and lower limbs (Table 1). For patient 5, who had numbness and a positive pinprick test, the sensory nerve action potential (SNAP) and sensory conduction velocity (SCV) in the median, ulnar, and sural nerves were normal. We further performed sympathetic skin response (SSR) and contact heat evoked potential (CHEP) tests and found a prolonged latency in the SSR test (2,497 vs. 1,036 ms) and a prolonged latency (366 vs. 337 ms) and decreased amplitude (43.5 vs. 70.8 μV) in the CHEP test on the right side of the leg vs. the left. MRI of lumbar nerves (coronal 3D-STIR) showed normal morphology of lumbar nerve roots and the lumbar plexus. MRI of calf skeletal muscles (T2WI FS cor, fat fraction) demonstrated slight fatty infiltration and edema in the bilateral anterior tibial muscle and extensor hallucis longus muscle, as well as distinct fatty infiltration in the gastrocnemius muscles (Figure 2).

### *SORD* Mutation Analysis

**Figure 3:**
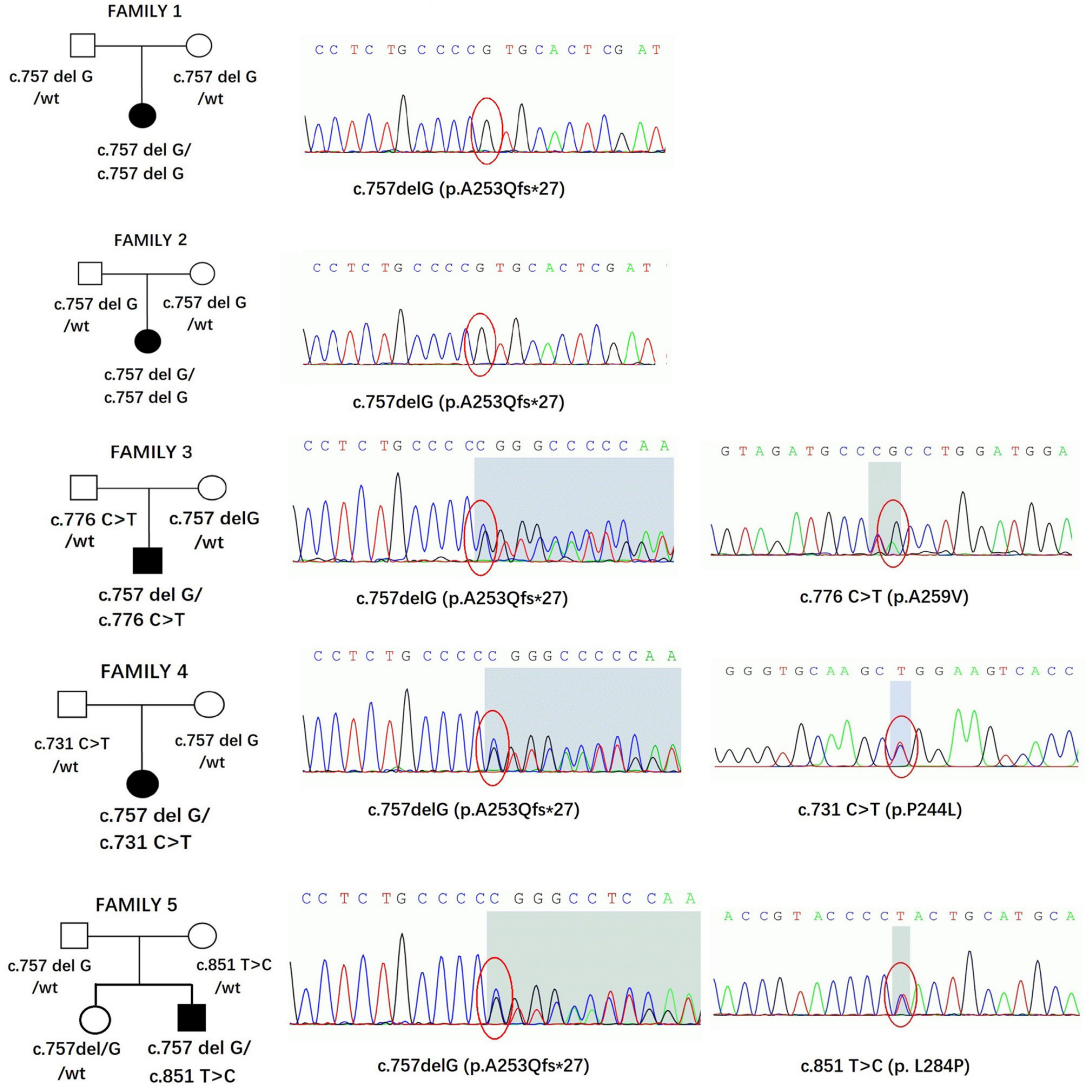
Pedigrees and genotypes of five families in our study with *SORD* variants. The chromatogram is from the affected patients.

Five affected patients were born to healthy nonconsanguineous parents. The pedigrees and genotypes of the families who carried the *SORD* variants are illustrated in Figure 3. Two individuals (patients 1 and 2) carried a previously reported homozygous c.757delG (p. A253Qfs^*^27) variant that was inherited from their parents separately. The frequency of c.757delG (p. A253Qfs^*^27) was 0.0046 (3/650) in our 650 healthy Chinese Han population. Based on the GnomAD v3 database, the allelic count of this variant is 623 out of 142,588, ranging from 0.002 in Asian populations to 0.005 in the European population and 0.007 in the Latin American population (4, 9). Only one homozygous variant encoding p. A253Qfs^*^27 has been detected in the East Asian population, but no homozygous mutation was found in our control subjects.

**Figure 4:**
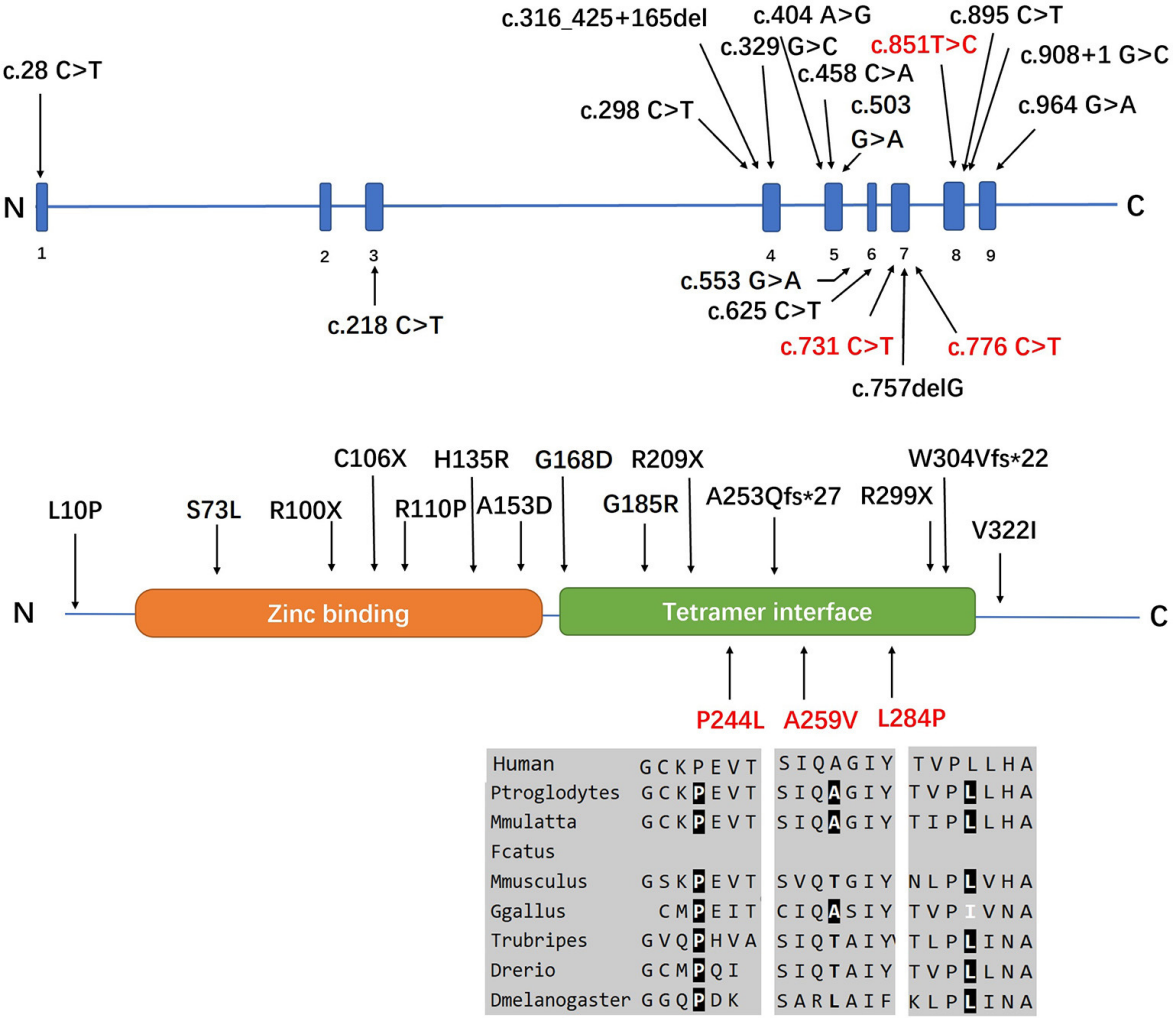
Positions of all the *SORD* mutations identified in the cases in this study. Three individuals (index patients from Families 3, 4, and 5) carried the heterozygous c.757delG (p. A253Q*Ter27) variant together with a second novel variant, including c.731 C>T (p.P244L), c.776 C>T (p.A259V), or c.851T>C (p.L284P). All variants had MAF <0.00001 in GnomAD 3.0 and were not observed in our database of 650 healthy controls. All novel missense variants are highly conserved across different species, with genomic evolutionary rate profiling (GERP) scores >3 (ranging from 4.74 to 5.29). The novel mutations identified in our study are marked in red.

Three individuals carried the heterozygous c.757delG (p. A253Qfs^*^27) variant together with a second novel variant, including c.731 C>T (p.P244L), c.776 C>T (p.A259V), or c.851T>C (p.L284P). All variants had MAF (minor allele frequency) <0.00001 in GnomAD 3.0 and were not observed in our database of 650 healthy controls. A bioinformatics tool was utilized to determine the pathogenicity of the *SORD* variants. All novel missense variants are highly conserved across different species, with genomic evolutionary rate profiling (GERP) scores >3 (ranging from 4.74 to 5.29). These variants are predicted to be disease causing in Mutation Taster. P. P244L and p. L284P are predicted to be damaging and probably damaging in SIFT and Polyphen, respectively, whereas p. A259V is predicted to be tolerated or benign. The novel variants are classified as likely pathogenic according to the standards and guidelines of the ACMG (8). The results of *in silico* analysis and pathogenicity prediction of these variants are provided in Table 2. The heterozygous mutation c.757del G was also detected in a healthy sister in Family 5, but c.851T>C was not detected (Figure 3). The locations and distributions of *SORD* variants in previous studies and our study are presented in Figure 4.

**Table 2:** Molecular analysis results and predicted pathogenicities of the variants in this study.

**Family**	**Exon**	**Nucleotide change**	**AA change**	**Database**			**Pathogenicity**			**GERP**	**Evidence**	**ACMG**	**Chromosomal location**	**HGMD/ClinVar**
				**GnomAD**	**1000 G**	**650 controls**	**SIFT**	**Polyphen-2**	**Mutation taster**				**(chr15)**	
1,2	7	c.757 del G	p.A253Qfs*27	0.0004149	0	0.004615						Pathogenic	45361217	HGMD
3	7	c.757 del G	p.A253Qfs*27	0.0004149	0	0.004615						Pathogenic	45361217	HGMD
	7	c.776 C>T	P. A259V	0.0000250	0	0	Tolerated	Benign	Disease causing	4.74	PM2+PM3+ PP1+PP3	Likely Pathogenic	45361240	Novel
4	7	c.757 del G	p.A253Qfs*27	0.0004149	0	0.004615	/	/				Pathogenic	45361221	HGMD
	7	c.731 C>T	p. P244L	0.0000108	0	0	Damaging	Most likely, damaging	Disease causing	4.74	PM2+PM3+ PP1+PP3	Likely Pathogenic	45361195	Novel
5	7	c.757 del G	p.A253Qfs*27	0.0004149	0	0.004615						Pathogenic	45361217	HGMD
	7	c.851 T>C	p. L284P	0	0	0	Damaging	Most likely, damaging	Disease causing	5.29	PM2+PM3+ PP1+PP3	Likely Pathogenic	45364579	Novel

*The novel likely pathogenic variants in SORD are marked in red*.

Currently, 11 biallelic mutations in *SORD* that are either pathogenic or likely to be pathogenic have been described in patients with CMT2 and dHMN. These cases are summarized in Table 3.

**Table 3:** Summary of all previously reported SORD-related CMT cases.

**References**	**Individuals/families**	**Genotype**		**Frequency**	**Family history**	**Age of onset**	**Phenotype**	**Weakness**	
		**Allele1**	**Allele2**					**Upper limb**	**Lower limb**
Cortese et al. (4) (*n* > 1,110)	45/38	c.757 del G	c.757 del G c.298 C>T c.329 G>C c.458 C>A c.964 G>A c.316_4251 + 65 del c.28 C>T c.895 C>T	>10%	14 /45 AR 7 Sporadic 31	17.2, SD 7.6 Range 2–40	CMT2 23 dHMN 18 CMT intermediate 4	26/44	43/44
Yuan et al. (5) (*n* = 215)	3/3	c.757 del G	c.757 del G c.625 C>T	1.39% (3/215) 7.5% (3/40 CMT2)	0/3 Sporadic 3	16, SD 1 Range 15–17	CMT2 3	0/3	3/3
Dong et al. (6) (*n* = 29)	4/4	c.757 del G c.404 A>G	c.757 del G c.9081 + G>C	13.8% (4/29)	0/4 Sporadic 4	12.5, SD 3.5 Range 9–16	dHMN 4	4/4	4/4
Lassuthova et al. (7) (*n* = 2,313)	18/16	c.757 del G	c.757 del G c.458 C>A c.218 C>T c.553 G>A c.503 G>A	0.78%(18/2313)	4/16 AR 4 Sporadic 12	15, SD 12.6 Range, 0–51	CMT2 14 dHMN 2 CMT intermediate2	NR	14/15
This study (*n* = 485)	5/5	c.757 del G	c.757 del G c.776 C>T c.731 C>T c.851 T>C	1% (5/485) 6.5% (5/78)	0/5 Sporadic 3	14, SD 4.5 Range 6–17	dHMN 5	2/5	5/5

*NR, not recorded*.

## Discussion

We identified four *SORD* variants from five unrelated patients in a cohort of 485 Chinese patients with hereditary neuropathy. The detection rate for biallelic *SORD* mutations or variants was 1% (5/485) in all patients with hereditary neuropathy and 6.41% (5/78) in patients with unclarified CMT2 and dHMN. These results were consistent with those from previous studies by members of the inherited neuropathies consortium (INC), which identified 45 individuals from 38 families in a cohort of over 1,000 families (4). Another recent study reported that the detection rate was 7.5% (3/40) in patients with CMT2 and 1.4% (3/215) in patients with CMT (5), while another study reported a detection rate of 13.8% (4/29) in patients with unclarified CMT2 and dHMN (6). Our findings provide valuable genetic and phenotypic information on the *SORD* gene in a large cohort of CMT and dHMN patients.

Our control database confirmed that the c.757delG (p. A253Qfs^*^27) variant was the most predominant mutation in the *SORD* gene. The carrier frequency was 0.0046 individuals in healthy controls (3/650). The frequency is slightly higher than the data reported in the GnomAD database, which described a frequency of 0.002 in Asian populations (9). No homozygous c.757delG (p. A253Qfs^*^r27) variant was found in our control database. Despite the relatively high frequency of the heterozygous c.757delG (p. A253Qfs^*^27) variant, the reason why *SORD* had not been previously identified as a causative gene for recessive CMT2 or dHMNs may be partly due to the presence of the pseudogene, which is a highly homologous, but nonfunctional, paralog of *SORD* (4).

All affected individuals with biallelic pathogenic mutations in *SORD* in this study presented with pure motor axonal neuropathy with childhood and adolescent onset, and the mean age of onset was 14 years. They shared a characteristic phenotype by symmetrical distal muscle wasting of the lower limbs. The progression rate was mild or modest, and all patients maintained their ability to walk. Two patients (patients 1 and 5) complained of numbness and episodic pain in the distal lower limb. The neurological examination showed hypersensitivity in the pinprick test, although the SNAP and SCV of the sensory nerve remained normal. Electrophysiological studies of patient 5 revealed a definite change in SSR and CHEP on the right side of the leg, indicating that small fiber impairment was also involved. Yuan et al. also reported two patients with c.757delG (p. A253Qfs^*^27) homozygous mutations in *SORD* who exhibited positive signs of dermographism. This may be attributed to the excitation of mechano-insensitive C-fibers on the axon, although dermographism is a common phenomenon without clear etiology. Sorbitol accumulation has been demonstrated to be related to diabetic polyneuropathy, which also has prominent C-fiber involvement (10–12). Therefore, if the patient had unexplained pain in *SORD*-related neuropathy, small fiber testing might be considered (13, 14).

*SORD* is an important enzyme that converts sorbitol to fructose in the two-step polyol pathway previously implicated in diabetic neuropathy. Cortese et al. found a complete loss of *SORD* protein and increased intracellular sorbitol in patient-derived fibroblasts, and the aldose reductase inhibitor epalrestat or ranirestat rescued the lack of *SORD* orthologs that caused synaptic degeneration and motor deficiency in *Drosophila* (4). However, the mechanism of axonal damage caused by *SORD* mutation has not been fully elucidated, and there are some questions that need to be further investigated: (1) The results of *SORD* deficiency are based on patient-derived fibroblasts with nonsense variants encoding homozygous p. A253Qfs^*^27 and compound heterozygous p. A253Qfs^*^27/p. R299Ter. However, the mechanism is unclear with regard to patients with missense mutations (p.L10P, p.R110P, p.H135R, p.A153D, p.V322I) in previous studies (4, 6) and those with the three missense mutations in our study (p.P244L, p. A259V, and p.L284P). Additional mechanisms, including increased levels of sorbitol and cellular osmolarity, oxidative stress, and decreased NADPH levels, are plausible (4, 15, 16). (2) Although patients with CMT2/dHMNs and *SORD* mutations share a similar mechanism and treatment to those with diabetic neuropathy, the clinical manifestations between motor CMT2 and diabetic neuropathy vary widely. Patients with diabetic neuropathy more commonly exhibit sensory and autonomic symptoms and develop numbness, tingling, and, in 20% of cases, neuropathic pain in the feet that progresses proximally toward the knees over time. In contrast, patients with *SORD* mutations often have affected motor neurons or motor nerves. Why mutations in ubiquitously expressed genes can cause different phenotypes of neuropathy remains unclear. (3) An animal model of C57BL/LiA mice with an intronic splicing *SORD* mutation exhibited decreased levels of *SORD* protein but did not develop neuropathy (17, 18). *Drosophila* has two functional *SORD* proteins that are 75 and 73% identical to the human *SORD* protein, and *Drosophila melanogaster* models of *SORD* deficiency could not fully mimic the pathogenesis of *SORD*-related neuropathy, especially sensory nerve impairment. Another animal model needs to be designed and used for research on the pathogenesis of *SORD*-related neuropathy.

In summary, the frequency of *SORD* mutations was calculated to be 6.4% in patients with unclarified CMT2 and dHMN. Our study further revealed a frequency of the c.757delG (p.A253Qfs^*^27) variant in the heterozygous state of 0.0046, and no homozygous variants were found in healthy Chinese Han controls. Three novel, likely pathogenic, variants of *SORD* (p.P244L, p.A259V, and p.L284P), together with p.A253Qfs^*^27, were identified in our study. The typical manifestation of *SORD*-related neuropathy is adolescent onset of predominantly motor involvement in the lower limb. Two cases with minor sensory involvement and a positive response to the pinprick test suggest that the C-fiber of the autonomic system may be affected, although the mechanism needs to be further investigated.

## Data Availability Statement

The original clinical and electrophysiological data presented in the study are included in the article, further inquiries can be directed to the corresponding author. The genetic information is available at https://www.ncbi.nlm.nih.gov/sra; Accession number: PRJNA764006.

## Ethics Statement

The studies involving human participants were reviewed and approved by the Ethics Committee of Peking University Third Hospital (IRB 00006761). Written informed consent to participate in this study was provided by the participants' legal guardian/next of kin. Written informed consent was obtained from the individual(s), and minor(s)' legal guardian/next of kin, for the publication of any potentially identifiable images or data included in this article.

## Author Contributions

XL and DF conceived and designed the study. XD and JH provided valuable clinical materials and wrote the paper. XL and MY performed the genetic testing. DF reviewed and edited the manuscript. All authors read and approved the manuscript.

## Funding

This study was supported by the Key R&D Plan of the Department of Science and Technology (No. XZ202001ZY0005G). This study was supported by the Peking University Clinical + X Youth Program (2021–2022; No. PKU2021LCXQ019).

## Conflict of Interest

The authors declare that the research was conducted in the absence of any commercial or financial relationships that could be construed as a potential conflict of interest. The reviewer YY declared a shared affiliation, with no collaboration, with the authors to the handling editor at the time of the review.

## Publisher's Note

All claims expressed in this article are solely those of the authors and do not necessarily represent those of their affiliated organizations, or those of the publisher, the editors and the reviewers. Any product that may be evaluated in this article, or claim that may be made by its manufacturer, is not guaranteed or endorsed by the publisher.
